# Protective Effect of *Liriope platyphylla* Root Extract on Dextran Sulfate Sodium-Induced Ulcerative Colitis in Mice

**DOI:** 10.4014/jmb.2512.12009

**Published:** 2026-05-04

**Authors:** You-Ree Nam, Hyeon-ji Lee, Bong-Hwan Park, Hae-Jin Jeong, Kyung-Ah Kim

**Affiliations:** 1 Department of Food and Nutrition, Chungnam National University, Daejeon 34134, Republic of Korea; 2 Glocal Life-care Convergence, Chungnam National University, Daejeon 34134, Republic of Korea

**Keywords:** *Liriope platyphylla* root, Dextran sulfate sodium, Ulcerative colitis, Inflammation, Disease activity index, Fecal bacteria

## Abstract

Ulcerative colitis (UC), a major form of inflammatory bowel disease, is characterized by chronic inflammation of the colon resulting from disruption of the intestinal barrier and excessive immune activation. Natural compounds with immunomodulatory properties and fewer adverse effects have therefore attracted attention as potential alternatives to conventional therapies. In this study, therapeutic effects of heat-treated *Liriope platyphylla* root extract (LP) were evaluated in a dextran sulfate sodium (DSS)-induced mouse model of colitis. Oral administration of LP (300 mg/kg/day) markedly ameliorated DSS-induced clinical symptoms, as evidenced by reduced body weight loss, lower disease activity index scores, and restoration of colon length. Histological examination confirmed that LP attenuated mucosal erosion, crypt damage, and inflammatory cell infiltration. LP administration suppressed the DSS-induced upregulation of pro-inflammatory cytokines (tumor necrosis factor-alpha, interleukin 6, interleukin 1 beta) and inflammatory enzymes (inducible nitric oxide synthase and cyclooxygenase 2) in both serum and colon tissues. Mechanistically, LP inhibited nuclear translocation of nuclear factor-kappa B (NF-κB) p65 and reduced the phosphorylation of mitogen-activated protein kinase (MAPK) molecules (extracellular signal-regulated kinase, c-jun n-terminal kinase, and p38), indicating suppression of key inflammatory signaling pathways. Moreover, LP helped restore gut microbial balance by reducing pathogenic *Enterobacteriaceae* and partially increasing beneficial *Bifidobacterium* populations. Taken together, these findings suggest that heat-treated LP alleviates colitis through the suppression of inflammatory mediators, inhibition of NF-κB and MAPK pathway activation, and modulation of gut microbiota. Therefore, LP may represent a promising natural therapeutic candidate for the management of colonic inflammation.

## Introduction

Inflammatory bowel disease (IBD) is a chronic, recurring, inflammatory disorder of the gastrointestinal tract, mainly comprising ulcerative colitis (UC) and Crohn’s disease (CD) [1, 2]. Although CD is a transmural inflammatory condition that can affect any part of the gastrointestinal tract, UC is characterized by continuous mucosal inflammation confined to the colon and rectum, and typically presents with abdominal pain, rectal bleeding, diarrhea, and weight loss [3, 4]. The global incidence and prevalence of UC have been steadily increasing, particularly in newly industrialized regions in Asia and Latin America, where dietary and environmental changes associated with Westernization are occurring [1, 5]. At the molecular level, UC pathogenesis involves disruption of the intestinal epithelial barrier and excessive immune responses, with elevated levels of proinflammatory cytokines such as tumor necrosis factor-alpha (TNF-α), interleukin 6 (IL-6), and interleukin 1 beta (IL-1β) [2, 6]. A compromised mucosal barrier leads to increased permeability and microbial translocation of these inflammatory mediators, activating intracellular signaling cascades such as the nuclear factor-kappa B (NF-κB) and mitogen-activated protein kinase (MAPK) pathways, ultimately leading to epithelial damage and ulcer formation [4, 7, 8].

Currently approved treatments for UC include aminosalicylates, corticosteroids, immunosuppressants such as azathioprine, and biologics targeting TNF-α, such as infliximab [9-11]. Although effective in inducing remission, these drugs can cause serious side effects, including immunosuppression, hepatotoxicity, and infections [12, 13]. Moreover, corticosteroids are ineffective for long-term maintenance therapy and are associated with high relapse and colectomy rates [13, 14]. Consequently, there is growing interest in naturally derived agents that provide reliable efficacy while minimizing adverse effects [7, 15].

*Liriope platyphylla*, a perennial plant belonging to the lily family, has long been used in traditional East Asian medicine to treat chronic cough, asthma, phlegm, and inflammatory diseases [16-18]. The therapeutic effects of *Liriope platyphylla* water extract have been reported in NC/Nga mice with atopic dermatitis [18]. In an asthma mouse model, *Liriope platyphylla* was found to suppress airway inflammation and hyper-responsiveness, and to act as an immunomodulator by regulating the imbalance between Th1/Th2 cytokines [16]. In addition, spicatoside A, a compound isolated from *Liriope platyphylla*, promoted neurite outgrowth in rat PC12 pheochromocytoma cells via activation of the tyrosine kinase A receptor pathway [19]. In a mouse macrophage model, root extracts of *Liriope platyphylla* containing steroidal saponins such as spicatoside A and ophiopogonin D have been shown to inhibit inflammatory mediators, thereby exhibiting immunomodulatory and anti-inflammatory effects [20]. Ethanol extract of heat-treated *Liriope platyphylla* was reported to inhibit MAPK pathway activation and to downregulate the expression of inflammatory cytokines in lipopolysaccharide-stimulated RAW264.7 macrophages [21]. The effects of heat-treated *Liriope platyphylla* extract were evaluated using a dextran sulfate sodium (DSS)-induced mouse colitis model. Among the cultivated varieties, the “Cheongsim” cultivar has been selectively developed and grown in Chungcheongnam-do, Republic of Korea. Comparative metabolomic analysis demonstrated significant quantitative differences in steroidal saponins, phenolic compounds, and primary metabolites, including spicatoside A, between Cheongsim and other cultivars [22]. Nutritional and physiological characterization further demonstrated variations in carbohydrate composition, amino acid profiles, and selected biofunctional activities between Cheongsim and cultivars such as Liriope Tuber No. 1 [23]. These findings suggest that Cheongsim is a chemically distinct cultivar that may serve as a standardized source for functional and experimental studies. Based on these cultivar-specific characteristics, the anti-inflammatory effects of heat-treated Cheongsim-derived *Liriope platyphylla* extract were subsequently assessed in the DSS-induced colitis model.

## Materials and Methods

### Reagents and Chemicals

Dextran sulfate sodium (DSS, 36–50 kDa) was purchased from MP BioMedicals (USA). Bradford Protein Assay Dye Reagent was purchased from Bio-Rad (USA). Enzyme-linked immunosorbent Assay (ELISA) kits for TNF-α, IL-1β, and IL-6 were obtained from R&D Systems (USA). RNAiso Plus was obtained from Takara Bio Inc., (Japan). BioFACT™ RT-Kit and BioFACT™ 2X Real-Time PCR Master Mix for quantitative real-time PCR (qRT-PCR) were obtained from BioFACT™ (Republic of Korea). RIPA lysis buffer, protease, and phosphatase inhibitors for protein extraction were purchased from Thermo Fisher Scientific (USA). Bovine serum albumin (BSA) was obtained from Millipore (Probumin Millipore, USA). Primary antibodies against cyclooxygenase (COX)-2, inducible NO synthase (iNOS), β-actin, p65, Lamin B1, p38, phospho-p38 (p-p38), ERK, phospho-ERK (p-ERK), JNK, and phospho-JNK (p-JNK), and secondary antibodies were obtained from Cell Signaling Technology (USA). Enhanced chemiluminescence (ECL) solution was obtained from ATTO (EzWestLumi plus, Japan). Desoxycholate (DHL) agar, BL agar, and sheep blood for gut microbiota analysis were purchased from Kisan (Kisan Bio, Republic of Korea).

### Sample Preparation

The Cheongsim cultivar of *Liriope platyphylla*, selectively cultivated in Chungcheongnam-do, Republic of Korea, was used in this study. Cheongsim has previously been characterized through comparative metabolic and nutritional analyses, which revealed cultivar-specific differences in steroidal saponins and primary metabolites [22, 23]. Plant materials were provided by the Goji Research Institute of the Chungcheongnam-do Agricultural Research and Extension Services via the Elohim Research Institute for experimental use. The heat treatment of *Liriope platyphylla* roots (LP) was performed by roasting at 250°C for 20 min using a Roaster (Samsung food machine, Republic of Korea). The roasted roots were then extracted once in 60% ethanol at 70°C for 6 hours, with the extraction vessel shaken and stirred every hour. The resulting extract was filtered through filter paper and concentrated under reduced pressure at 35°C using a rotary evaporator (EYELA, Japan).

### Mice and Experimental Design

**Fig. 1:**
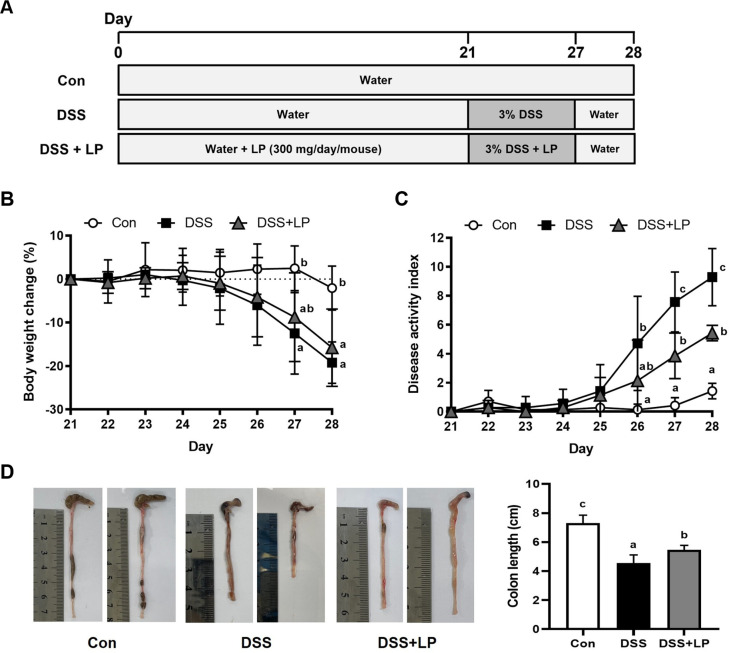
Effects of LP extract on the clinical symptoms of DSS-induced colitis. (**A**) Schema of the DSS-induced mice model. (**B**) Changes in body weight during the experiment period. (**C**) Disease activity index (DAI). (**D**) Representative images and measurement of colon length. Data are expressed as the mean ± SD (n = 7 per group). Means with different letters indicate statistically significant differences among groups (*p* < 0.05) as determined by Tukey’s multiple comparison test. DSS, dextran sulfate sodium; SD, standard deviation.

Male C57BL/6N mice (8 weeks old) were purchased from Orient Bio (Republic of Korea) and maintained under controlled environmental conditions (12 hours light/dark cycle, 22 ± 2°C, and humidity 50 ± 10%) with ad libitum access to a standard laboratory diet and tap water. The mice were randomly divided into three groups: control (Con), 3% DSS-induced colitis (DSS), and DSS with LP treatment (DSS + LP). Each experimental group consisted of seven mice (n = 7). The experimental design is illustrated in Fig. 1A. LP was administered orally by gavage to the DSS + LP group at a dose of 300 mg/kg/day for 27 consecutive days, a schedule designed to assess its protective effects both prior to and during DSS-induced colitis. Colitis was induced by providing 3% DSS in the drinking water to the DSS and DSS + LP groups from days 21 to 27 (Fig. 1A). On day 28, mice were euthanized by an overdose of isoflurane inhalation, and blood and tissue samples were immediately collected. Colon length was measured, and samples were either fixed in 10% neutral formalin for histological evaluation, used for mRNA analysis, or snap-frozen in liquid nitrogen and stored at -80°C until further use.

All animal procedures were performed in accordance with the guidelines of the Institutional Animal Care and Use Committee (IACUC) of Chungnam National University (Approval No. 202409A-CNU-161).

### Disease Activity Index (DAI) and Colon Length Measurement

Colitis symptoms were monitored daily using the Disease activity index (DAI) during the 7 days of DSS administration. The DAI score was calculated as the mean of the weight loss, stool consistency, and stool occult blood scores [24]. The scores were as follows: weight loss (0, none; 1, 1-5%; 2, 5-10%; 3, >10%); stool consistency (0, normal; 1, slightly watery stool; 2, watery stool; 3, watery diarrhea); and stool occult blood (0, none; 1, slightly bloody stool; 2, bloody stool; 3, severe bleeding).

### ELISA for Pro-Inflammatory Factor Levels

Blood samples from the inferior vena cava of mice were collected in serum separation tubes. Serum was collected by centrifugation of whole blood samples at 4,000 × g for 10 min and stored at -80°C until analysis. Colon tissue and lysis buffer were added to bead tubes and homogenized using a Benchmark BeadBlaster (Benchmark Scientific, USA) at 4°C. The homogenate was centrifuged at 14,000 × g for 10 minutes at 4°C, and the supernatant was collected for analysis. Pro-inflammatory cytokines (TNF-α, IL-1β, and IL-6) in the isolated supernatant and serum were quantified using ELISA kits according to the manufacturer's procedure.

### Histological Analyses

Colon tissues were fixed in 10% neutral formalin, and hematoxylin-eosin (H&E) staining was performed at the Korean Pathology Technical Center (KP&T, Republic of Korea). Colon slides were examined using a digital microscope (Mateo FL, Leica Microsystems, Germany). Histological damage in colon tissue was assessed using a scoring system evaluating epithelial loss (0–3), crypt damage (0–3), goblet cell depletion (0–3), and infiltration of inflammatory cells (0–3) [25].

### Analysis of Fecal Bacteria

Prior to euthanasia, fecal samples (0.1 g) were collected from each group and homogenized in 0.9 mL of sterile phosphate-buffered saline. The homogenates were serially diluted 10-fold and plated onto selective agar media. *Enterobacteriaceae* were cultured aerobically on DHL agar at 37°C for 24 hours, whereas *Bifidobacterium* were cultured anaerobically on BL agar at 37°C for 48 hours. This analysis was conducted following a modified method described by Kim *et al*. (2012) [26].

### Quantitative Real-Time Polymerase Chain Reaction (qRT-PCR) Analysis

Total RNA was extracted using the RNAiso Plus kit, and reverse transcription into cDNA was performed using the BioFACT™ RT-Kit. qRT-PCR was performed using the BioFACT™ 2X Real-Time PCR Master Mix and the QuantStudio™ 3 Real-Time PCR System (Applied Biosystems, USA). The relative expression of target genes was determined using the 2^−ΔΔCt^ method and normalized to the expression of the internal reference Gapdh. Table 1 shows the primers used in qRT-PCR.

### Western Blotting

Colon tissues were homogenized with RIPA lysis buffer containing protease and phosphatase inhibitors. Protein concentrations were determined using the Bradford assay. Equal amounts of protein were separated by SDS-PAGE and transferred onto polyvinylidene fluoride (PVDF) membranes. For subcellular fractionation, nuclear and cytosolic proteins were isolated using a commercial Nuclear Extraction kit (Abcam, USA) according to the manufacturer’s instructions. The purity of each fraction was verified using Lamin B1 as a nuclear marker and β-actin as a cytosolic marker. Membranes were blocked with 3% bovine serum albumin (BSA) in Tris-buffered saline containing Tween 20 (TBST) for 1 h at room temperature and incubated with primary antibodies overnight at 4°C, followed by incubation with secondary antibodies for 2 h at room temperature. Protein bands were detected using enhanced chemiluminescence (ECL) reagents and visualized using a LuminoGraph I imaging system (ATTO, Japan). Densitometric analysis was performed using ImageJ software (National Institutes of Health, USA) with samples derived from independent biological replicates (n = 7 per group). Phosphorylated MAPK proteins were normalized to their respective total protein levels (p-ERK/ERK, p-JNK/JNK, p-p38/p38), while nuclear and cytosolic proteins were normalized to Lamin B1 and β-actin, respectively.

### Statistical Analysis

Data are presented as the mean ± standard deviation (SD). Body weight change and Disease activity index (DAI) data were analyzed using repeated-measures analysis of variance (RM-ANOVA), while all other data were evaluated using one-way analysis of variance (ANOVA) followed by Tukey’s multiple comparison test. All statistical analyses were performed using IBM SPSS Statistics 26 (IBM Corp., USA), and differences were considered statistically significant at *p* < 0.05. Values with different superscripts (a–c) indicate significant differences among groups.

## Results and Discussion

### Effects of LP on Clinical Symptoms and Colon Damage in DSS-Induced Colitis

The addition of DSS to drinking water induces either acute or chronic colitis, depending on the treatment protocol, with weight loss being the most prominent symptom [27]. The DSS-induced colitis model produces symptoms such as weight loss, loose stools, or diarrhea, and sometimes evidence of rectal bleeding [28]. To assess the effects of LP on clinical symptoms and colonic damage in DSS-induced colitis, body weight, DAI scores, and colon length were evaluated. Mice treated with DSS alone exhibited substantial weight loss compared with those of the Con group, whereas LP administration partially attenuated this weight loss, although statistical significance was not consistently observed (Fig. 1B). The DAI score, which integrates parameters such as body weight loss, stool consistency, and fecal bleeding, indicated a marked increase in colitis severity in the DSS group relative to controls (Fig. 1C). However, treatment with LP extract markedly reduced DAI scores compared with those of the DSS group. In the DSS group, which exhibited moderate UC symptoms, the colon length was substantially reduced compared with those of the Con group (Fig. 1D), consistent with the well-established use of colon shortening as an indicator of colitis severity [24, 29]. The colon length of the LP group increased markedly compared with that of the DSS group (Fig. 1D). Collectively, these findings suggest that LP effectively protects against DSS-induced clinical symptoms and colon damage.

### Effects of LP on Histological Changes in the Colon Tissue in DSS-Induced Colitis

**Fig. 2:**
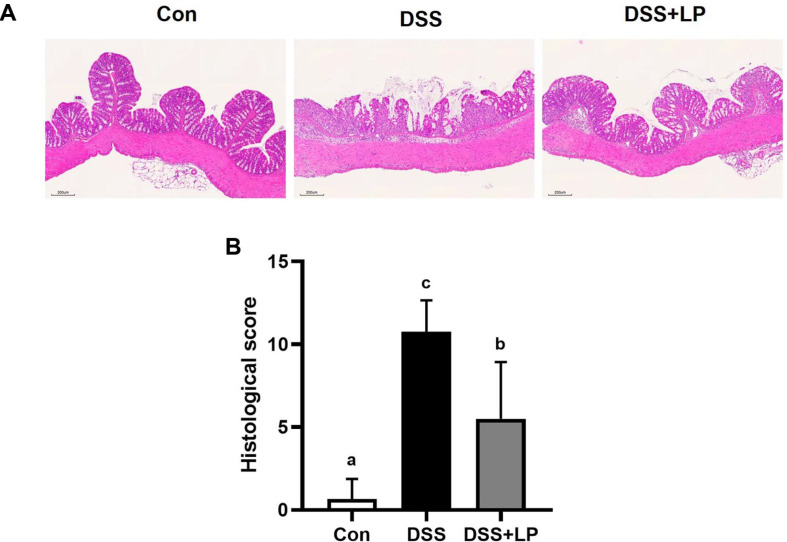
Effects of LP extract on colon damage of DSS-induced colitis mice. (**A**) Representative images of microscopic colon tissue stained with H&E (Original magnification X 200). (**B**) Histology scores of each group. Data are expressed as the mean ± SD (n = 7 per group). Means with different letters indicate statistically significant differences among groups (*p* < 0.05) as determined by Tukey’s multiple comparison test. H&E, hematoxylin-eosin; SD, standard deviation; DSS, dextran sulfate sodium.

The colon tissue sections were stained with hematoxylin-eosin and evaluated using a histopathological scoring system to determine the extent of tissue damage (Fig. 2A and 2B). Severe inflammation and mucosal injury were observed in the DSS group, consistent with acute colitis. Sections from DSS-treated mice exhibited edema, infiltration of inflammatory cells, and extensive mucosal layer damage, with glandular structures largely disrupted (Fig. 2A), in agreement with previous reports [30, 31]. These changes were reflected in the histological damage scores, which were markedly elevated in the DSS group and substantially reduced in the LP-treated group (Fig. 2B). Administration of LP mitigated DSS-induced mucosal damage and inflammatory cell infiltration, leading to a significant decrease in histological damage scores.

### Effects of LP on the Levels of Pro-Inﬂammatory Cytokines in the Serum and Colon Tissue in DSS-Induced Colitis

Elevated levels of pro-inflammatory cytokines are one of the hallmark features of IBD [32]. In previous studies, elevated serum TNF-α levels in the DSS-induced colitis model reflected a systemic inflammatory response that increased intestinal permeability and enhanced mucosal damage [33]. Similarly, IL-6 levels were also upregulated in colonic tissue, promoting Th17 cell differentiation and maintaining an inflammatory microenvironment, thereby contributing to chronic inflammation [34]. The anti-inflammatory effects of LP in DSS-induced colitis were assessed by measuring serum TNF-α levels and the expression of pro-inflammatory cytokines (TNF-α, IL-6, and IL-1β) in colon tissue (Table 2). Serum TNF-α levels were significantly increased in the DSS group compared with those of the Con group, but were significantly reduced following LP administration (*p* < 0.05). In colonic tissue, IL-6 levels were also significantly elevated in the DSS group and significantly decreased in the LP-treated group (*p* < 0.05). Although IL-1β levels were markedly increased in the DSS group, LP treatment did not produce a statistically significant reduction. Similarly, TNF-α levels in colonic tissue tended to decrease after LP administration but did not reach statistical significance. Therefore, these results suggest that LP administration alleviates DSS-induced colitis primarily by reducing systemic inflammation and partially modulating mucosal cytokine responses. This is consistent with previous reports on the immunosuppressive and anti-inflammatory properties of LP-derived saponins such as spicatoside A and ophiopogonin D [20, 22].

### LP Modulated Inflammatory and Tight Junction–Related Gene Expression in Colon Tissues of DSS-Induced Colitis Mice

**Fig. 3:**
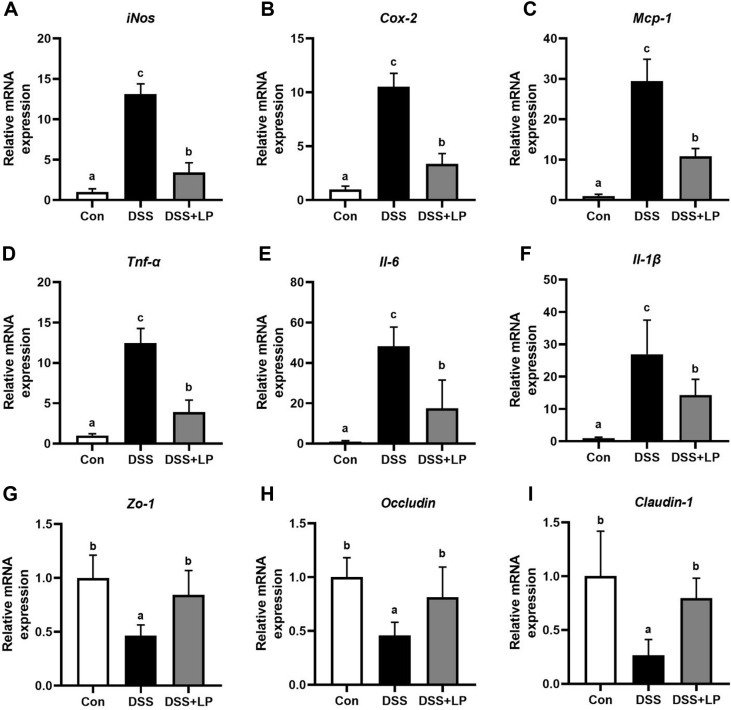
Effects of LP on inflammatory mediator and tight junction–related gene expression in colon tissues of DSS-induced colitis mice. mRNA levels of (**A**) *iNos*, (**B**) *Cox-2*, (**C**) *Mcp-1*, (**D**) *Tnf-α*, (**E**) *Il-6*, (**F**) *Il-1β*, (**G**) *Zo-1*, (**H**) *Occludin*, and (**I**) *Claudin-1* in colon tissues. Data are expressed as the mean ± SD (n = 7 per group). Means with different letters indicate statistically significant differences among groups (*p* < 0.05) as determined by Tukey’s multiple comparison test. DSS, dextran sulfate sodium; iNos, inducible nitric oxide synthase; Cox-2, cyclooxygenase-2; Mcp-1, monocyte chemoattractant protein-1; Tnf-α, tumor necrosis factor-alpha; Il-6, interleukin-6; Il-1β, interleukin-1 beta; Zo-1, zonula occludens-1; SD, standard deviation.

The mRNA expression levels of major inflammatory markers and tight junction-related genes in the colon tissue were investigated to evaluate the protective effect of LP against colitis in DSS-induced colitis mice (Fig. 3). The DSS group showed a marked increase in *iNos*, *Cox-2*, *Tnf-α*, *Il-1β*, *Il-6*, and *Mcp-1* expression compared with that of the Con group, indicating a strong transcriptional inflammatory response (Fig. 3A-3F). In contrast, treatment with LP downregulated the expression of all six genes. The expression of *iNos* and *Cox-2*, known markers associated with nitric oxide and prostaglandin synthesis, respectively, was markedly suppressed in the LP group. Similarly, the elevated transcription of pro-inflammatory cytokines, such as *Tnf-α*, *Il-1β*, and *Il-6* in DSS-induced mice was markedly reduced by LP treatment. These cytokines are critical regulators of colonic inflammation, contributing to immune cell recruitment, epithelial barrier disruption, and disease progression in IBD [32]. The attenuation of *Mcp-1* expression, a chemokine responsible for monocyte infiltration into inflamed tissues, supports the anti-inflammatory effect of LP.

In addition to inflammatory mediators, the expression of epithelial barrier-related genes was also evaluated. DSS treatment significantly decreased the mRNA expression of the tight junction proteins *Zo-1*, *Occludin*, and *Claudin-1*, indicating disruption of epithelial barrier integrity. Tight junction proteins play a crucial role in maintaining intestinal epithelial barrier function, and their downregulation is commonly observed in DSS-induced colitis models [33, 34]. The expression levels in the LP-treated group were comparable to those observed in the Con group. These findings suggest that LP treatment may contribute to maintaining epithelial barrier-related gene expression during DSS-induced intestinal inflammation.

Overall, these results indicate that LP administration attenuated DSS-induced transcriptional upregulation of inflammatory mediators while restoring the expression of tight junction–related genes in colon tissues. These findings are consistent with previous reports showing that plant-derived compounds containing steroid saponins can suppress cytokine gene expression under inflammatory conditions. Notably, LP is rich in spicatoside A, a member of the steroid saponin family [35, 36].

### Effects of LP on the NF-κB and MAPK Signaling Pathway in DSS-Induced Colitis

**Fig. 4:**
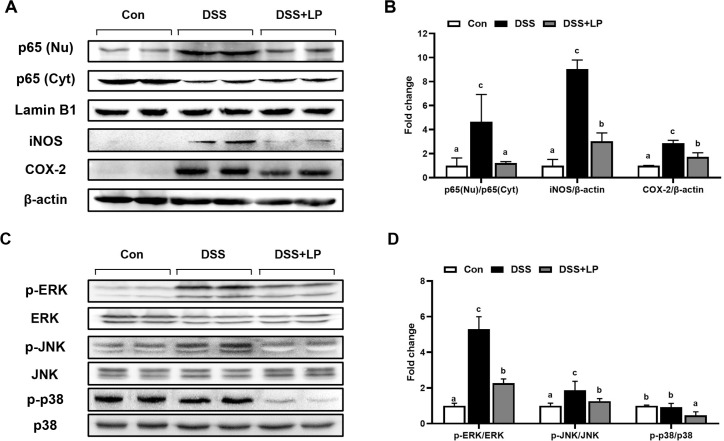
LP extract regulates the NF-κB and ERK/JNK signaling pathway in mice with DSS-induced colitis. (**A**) Expressions of NF-κB signaling pathway-related proteins p65 (Nu), p65 (Cyt), iNOS, and COX-2) in colon tissues. (**B**) Relative protein contents of p65 (Nu), p65 (Cyt), iNOS, and COX-2. (**C**) Expressions of MAPK signaling pathway-related proteins ((p-ERK, ERK, p-JNK, JNK, p-p38, and p38) in colon tissues. (**D**) Relative protein contents of p-ERK, ERK, p-JNK, JNK, p-p38, and p38. Data are expressed as the mean ± SD (n = 7 per group). Means with different letters indicate statistically significant differences among groups (*p* < 0.05) as determined by Tukey’s multiple comparison test. Nu, nucleus; Cyt, cytoplasm; DSS, dextran sulfate sodium; SD, standard deviation; NF-κB, nuclear factor kappa-light-chain-enhancer of activated b cells; ERK, extracellular signal-regulated kinase; JNK, c-jun n-terminal kinase; iNOS, inducible nitric oxide synthase; COX-2, cyclooxygenase-2; MAPK, mitogen-activated protein kinase.

The molecular mechanism underlying the anti-inflammatory effects of LP was elucidated by evaluating the expression and phosphorylation of key signaling proteins involved in the NF-κB and MAPK pathways, as well as the expression of iNOS and COX-2 proteins, in colon tissue from mice with DSS-induced colitis. The NF-κB pathway is a key regulator of inflammatory gene transcription. When activated, IκBα is phosphorylated and degraded, permitting the p65/p50 complex to translocate to the nucleus and induce the transcription of inflammatory genes [37]. In addition, p65 is phosphorylated at specific serine residues, enhancing transcriptional activity and the recruitment of coactivators, thereby amplifying the expression of inflammatory genes [38]. The intracellular localization of p65 protein was evaluated in both nuclear and cytosolic fractions (Fig. 4A). DSS treatment increased the nuclear translocation of p65, as evidenced by increased p65 levels in the nucleus and decreased p65 levels in the cytosol. In contrast, LP treatment markedly reversed this translocation pattern, reducing p65 levels in the nucleus and restoring p65 levels in the cytoplasm (Fig. 4B). These findings suggest that LP treatment was associated with reduced nuclear translocation of NF-κB p65 in colon tissues of DSS-induced colitis mice. This is consistent with previous reports that Rosmarinic acid and Astragalin inhibited DSS-induced nuclear translocation of NF-κB p65 and reduced the expression of inflammatory mediators [39, 40].

The iNOS catalyzes the production of nitric oxide, a reactive mediator involved in oxidative stress and mucosal damage, whereas COX-2 is responsible for the synthesis of prostaglandins, which promote inflammation and edema [41, 42]. In the current study, DSS-induced colitis substantially upregulated the expression of iNOS and COX-2 in the colon (Fig. 4A and 4B), whereas LP treatment markedly suppressed their expression, suggesting that LP treatment was associated with reduced expression of inflammatory mediators that are commonly regulated by NF-κB signaling. The suppression of iNOS and COX-2 expression has been reported to alleviate inflammation in experimental colitis models. The MAPK pathway, consisting of extracellular signal-regulated kinase (ERK), c-jun n-terminal kinase (JNK), and p38, mediates cellular responses to stress and inflammatory cytokines by activating transcription factors that regulate the expression of inflammatory genes [43]. DSS-induced colitis increased phosphorylation of MAPK-related proteins, especially ERK and JNK, whereas total protein levels were unchanged (Fig. 4C and 4D). Abnormal activation of the MAPK signaling pathway in IBD promotes the production of inflammatory cytokines and contributes to mucosal damage and disease progression [44]. LP administration significantly attenuated the phosphorylation of ERK and JNK, whereas changes in p38 phosphorylation were less pronounced. These results indicate that LP treatment was associated with reduced activation of ERK and JNK signaling pathways in DSS-induced colitis (Fig. 4C and 4D). Previous studies have reported activation of p38 MAPK signaling in IBD and experimental colitis models, including DSS-induced colitis [44]. However, the involvement of p38 signaling may vary depending on experimental conditions and cell types. For example, activation of p38 MAPK has been observed in intestinal immune cells during experimental colitis, whereas pharmacological inhibition of p38 has also been reported to aggravate DSS-induced colitis in some models [45]. These findings suggest that the role of p38 signaling in intestinal inflammation may be context-dependent. Consistent with these reports, the present study demonstrated greater modulation of ERK and JNK phosphorylation than of p38. The MAPK pathway plays a crucial role in regulating the expression of inflammatory cytokines and mediators, such as TNF-α, IL-6, and IL-1β, in IBD [32]. Although LP treatment was associated with reduced NF-κB nuclear translocation and MAPK phosphorylation, these signaling changes may partly reflect the overall reduction of intestinal inflammation. Further mechanistic studies will be necessary to determine whether LP directly regulates these signaling pathways.

### Effects of LP on Fecal *Enterobacteriaceae* and *Bifidobacterium* Counts in DSS-Induced Colitis

To assess whether LP modulates specific fecal bacterial populations in DSS-induced colitis, fecal bacterial counts were analyzed in mice. DSS administration disrupted the intestinal microbiota, causing a marked reduction in beneficial bacteria such as *Bifidobacterium* and an increase in potentially pathogenic *Enterobacteriaceae* (Table 3). Consistent with a previous report [31], the reduction in *Lactobacillus* and *Bifidobacterium* observed in the DSS group reflected the loss of protective commensals known to maintain mucosal immunity and suppress inflammatory responses. LP administration tended to increase *Bifidobacterium* counts compared with those of the DSS group, although the difference was not statistically significant. *Enterobacteriaceae* counts were markedly elevated in the DSS group, reflecting the bloom of pathobionts commonly associated with IBD [46]. LP treatment suppressed the overgrowth of *Enterobacteriaceae* compared with that of the DSS group (*p* < 0.05). These findings suggest that LP administration may contribute to the alleviation of DSS-induced colitis not only through anti-inflammatory signaling but also through modulation of selected fecal bacterial populations, including *Enterobacteriaceae* and *Bifidobacterium*.

Although LP demonstrated protective effects in the DSS-induced colitis model, the present study evaluated only a single administration dose (300 mg/kg/day). As a result, the dose-response relationship and minimal effective concentration of LP remain undetermined. Further studies incorporating multiple dosage levels are needed to define the optimal dose range and more comprehensively characterize its pharmacological efficacy. Another limitation is the lack of direct phytochemical characterization of the LP extract. While previous metabolomic studies have reported cultivar-dependent differences in major metabolites of *Liriope platyphylla*, including steroidal saponins and phenolic compounds, the specific composition of the extract used here was not analyzed [22]. Future studies will include chromatographic analyses (*e.g.*, high-performance liquid chromatography or liquid chromatography–mass spectrometry) to quantify bioactive compounds and standardize the extract.

In conclusion, heat-treated *Liriope platyphylla* alleviated DSS-induced colitis in mice by attenuating clinical symptoms, preserving colon length, and reducing histological damage. LP substantially suppressed the expression of pro-inflammatory cytokines and enzymes, accompanied by reduced NF-κB nuclear translocation and MAPK phosphorylation in colon tissues. These molecular effects contributed to the downregulation of inflammatory mediators, including TNF-α, IL-6, IL-1β, iNOS, and COX-2. Moreover, LP may mitigate colitis through modulation of inflammatory signaling pathways and selective alterations in fecal bacterial populations, such as *Enterobacteriaceae* and *Bifidobacterium*. Overall, these findings support the potential of LP as a natural anti-inflammatory agent for the management of colonic inflammation.

## Figures and Tables

**Table 1:** Primer sequences for qRT-PCR.

Primer name	Forward Primer sequence (5’-3’)	Reverse Primer sequence (5’-3’)
*iNos*	ATGTCCGAAGCAAACATCAC	TAATGTCCAGGAAGTAGGTG
*Cox-2*	GTGGAAAAACCTCGTCCAGA	TGATGGTGGCTGTTTTGGTA
*Tnf-α*	CCCCAAAGGGATGAGAAGTT	CACTTGGTGGTTTGCTACGA
*Il-6*	CCGGAGAGGAGACTTCACAG	CAGAATTGCCATTGCACAAC
*Il-1β*	GGATGAGGACATGAGCACCT	AGCTCATATGGGTCCGACAG
*Mcp-1*	GGAAAAATGGATCCACACCTTGC	TCTCTTCCTCCACCACCATGCAG
*Zo-1*	CCAGCAACTTTCAGACCACC	TTGTGTACGGCTTTGGTGTG
*Occludin*	TAAGAGCTTACAGGCAGAACTAG	CTGTCATAATCTCCCACCATC
*Claudin-1*	TCTGGCAAGTCTAGCAGTTTGTG	GCTAAGCTGCTAACCCTGTGGT
*Gapdh*	ACAACTTTGGCATTGTGGAA	GATGCAGGGATGATGTTCTG

**Table 2:** Effects of LP extract on serum and colon tissue cytokine levels of TNF-α, IL-6, and IL-1β in DSS-induced colitis mice.

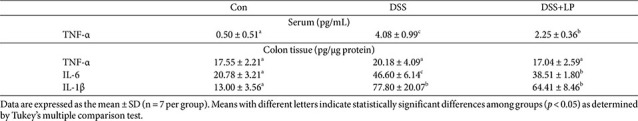

**Table 3:** Fecal *Enterobacteriaceae* and *Bifidobacterium* counts in DSS-induced colitis mice

	Con	DSS	DSS+LP
*Bifidobacterium* (1 × 10^5^ CFU/mL)	42.17 ± 20.21^b^	1.91 ± 1.32^a^	11.61 ± 12.25^a^
*Enterobacteriaceae* (1 × 10^5^ CFU/mL)	0.93 ± 1.16^a^	76.50 ± 19.90^b^	18.14 ± 22.45^a^

Data are expressed as the mean ± SD (n = 7 per group). Means with different letters indicate statistically significant differences among groups (*p* < 0.05) as determined by Tukey’s multiple comparison test.
